# An Intersectional Perspective on Digital Health: Longitudinal Narratives and Observations With Older and Middle-Aged Women Experiencing Homelessness

**DOI:** 10.1093/geront/gnaf021

**Published:** 2025-01-27

**Authors:** Sophie Nadia Gaber, Elisabet Mattsson, Anna Klarare, Joanna Dawes, Penny Rapaport, Women’s Advisory Board for Inclusion Health

**Affiliations:** Department of Women’s and Children’s Health, Healthcare Services and e-Health, Uppsala University, Uppsala, Sweden; Division of Psychiatry, Faculty of Brain Sciences, University College London, London, England; Department of Women’s and Children’s Health, Healthcare Services and e-Health, Uppsala University, Uppsala, Sweden; Department of Healthcare Sciences, Marie Cederschiöld University College, Stockholm, Sweden; Department of Women’s and Children’s Health, Healthcare Services and e-Health, Uppsala University, Uppsala, Sweden; Department of Healthcare Sciences, Marie Cederschiöld University College, Stockholm, Sweden; Institute of Epidemiology and Health Care, UCL Collaborative Centre for Inclusion Health, University College London, London, England; Division of Psychiatry, Faculty of Brain Sciences, University College London, London, England; Ersta Möjlighet, Stockholm, Sweden

**Keywords:** digital divide, eHealth, homeless, qualitative research, Women’s health

## Abstract

**Background and Objectives:**

People experiencing homelessness and older people encounter barriers as health and social care services are increasingly delivered online, however, there is limited knowledge about how this relates to older and middle-aged women experiencing homelessness, especially those from minoritized and/or migrant communities. We aimed to explore how technology, including digital health, can help or hinder older and middle-aged women to navigate paths through and out of homelessness.

**Research Design and Methods:**

This 16-month qualitative longitudinal study utilized narrative interviews and participant observations with seven older and two middle-aged women experiencing homelessness, in London, England. Additionally, we observed interactions between the women experiencing homelessness and 2 information and communications technology class facilitators. We collected and analyzed data using a narrative, interpretative approach. An advisory board of women with lived experiences of homelessness supported the interpretation of findings and development of practice and policy recommendations.

**Results:**

We present our findings as 3 composite narrative vignettes co-constructed with the participants: (1) “No, I’m not taking this telephone appointment”; (2) “Technology doesn’t judge you”; and (3) “You have to be a digital person now.” The findings illuminate determinants of digital health equity related to aging, gender, and migration status among older and middle-aged women experiencing homelessness.

**Discussion and Implications:**

Using an intersectional lens, we provide recommendations about how to better align digital health to the needs of older and middle-aged women experiencing homelessness. The findings will inform intervention development.

## Background and objectives

Increasing digitalization of services has implications for supporting people experiencing homelessness and an aging population. Digital health refers to the use of digital technologies for maintaining and enhancing health and well-being through healthcare delivery, health information management, and social support (World Health Organization (WHO), 2021). People experiencing homelessness have higher rates of both premature mortality and morbidity, including chronic disease, mental illness, and substance use, compared to stably housed populations (Aldridge et al., 2018; Suh et al., 2022). Digital health has potential to ameliorate health disparities by increasing access to information and services for people with complex health and social care needs (National Health Service [NHS] England, 2023; Polillo et al., 2021; Richardson et al., 2022). Scoping reviews indicate that older adults use digital technologies for health promotion and disease prevention, however, future research should account for diverse populations of older adults (De Santis et al., 2023; Wilson et al., 2021).

Prior to the Coronavirus pandemic, older people with poor self-reported health, low socioeconomic status, and who belong to specific minoritized communities had limited access to digital health services (Husain et al., 2022). Nevertheless, provision of digital health care consultations (e.g., telephone or web-based) increased rapidly for all during the pandemic (Husain et al., 2022). Portable internet and phone access have been identified as potential tools for facilitating health-related communication and self-management (Heaslip, Green, et al., 2021; Polillo et al., 2021), social connectivity and support (Heaslip, Richer, et al., 2021), and access to essential services and housing resources (Raven et al., 2018; Zahir et al., 2023) for people experiencing homelessness.

People experiencing homelessness encounter personal (e.g., privacy concerns, theft, mistrust about data management) and practical barriers (e.g., limited credit, restricted access to charging points; Coetzer et al., 2024; Heaslip, Richer, et al., 2021) which can impede digital health services. Specifically, older people experiencing homelessness face barriers such as financial constraints, cognitive impairments, and low digital literacy, hindering access to digital health and social care services (Raven et al., 2018; Zahir et al., 2023).

Previous research on digital health lacks disaggregation by gender, sex, and other demographics, such as ethnicity and migration status (Figueroa et al., 2021), and studies among people experiencing homelessness have focused on quantifiable measures of digital access and intervention outcomes (Heaslip, Richer, et al., 2021; Raven et al., 2018). Approximately 80% of people experiencing homelessness have access to a mobile or smartphone, and ownership is comparable between genders (Heaslip, Richer, et al., 2021). However, older people experiencing homelessness have significantly lower smartphone ownership and internet access compared to older adults in the general population (Raven et al., 2018). Limited research exists on subjective experiences of digital health according to different pathways through and out of homelessness for women (Polillo et al., 2021), especially for older women experiencing homelessness from minoritized and/or migrant communities.

### Theoretical Resources

This study adopted an intersectional perspective on digital health, recognizing that a person’s experiences are shaped and influenced by multiple intersecting identities and social systems of power and influence which cannot be understood in isolated or discrete categories (Crenshaw, 1989). Our theoretical perspective acknowledges recent expansions on intersectionality, including *relations of inequality* across dynamic life-course trajectories (e.g., aging, homelessness, migration; Ma and Joshi, 2021), as well as criticisms that intersectionality has been appropriated by White feminism in European policy (Christoffersen, 2022) and should recognize heterogeneity and multiple health disparities among older members of the lesbian, gay, bisexual or pansexual, transgender, queer, or questioning (LGBTQ+) community and those from minoritized and/or migrant communities (Hand and Ihara, 2024). A narrative approach can be used to explore how people experience digital health disparities that occur *within* and *across* multiple and intersecting disadvantages, such as older age, lower socioeconomic status, and limited English proficiency (Husain et al., 2022). Thus, we refer to the participants as “women experiencing homelessness” while acknowledging the heterogeneity of this term, including multiple and intersecting identities and experiences, such as being older or middle-aged women from minoritized and/or migrant communities.

Richardson et al. (2022) propose a digital health equity framework that complements intersectionality, by elucidating intersections across various levels: *individual* (e.g., cultural identity and sociodemographic characteristics, literacy, technology access, attitudes to use), *interpersonal* (e.g., social networks, technology bias, patient-provider relationships), *community* (availability and delivery of services), and *societal* (e.g., digital policies, social norms, ideologies, and discrimination; Richardson et al., 2022). Thus, this narrative study’s departure point was individual women’s experiences of digital health, and through an intersectional lens, we also acknowledge intersections, tensions, and opportunities within and across other interpersonal, community and societal considerations of digital health.

This study specifically explored intersections among aging, gender, migration status, and homelessness. Aging is salient to homelessness because exposure to cumulative disadvantage, including chronic mental and physical illness, functional and cognitive impairment, and substance use, has been associated with “accelerated aging” (Brown et al., 2019; Mantell et al. 2023; Suh et al., 2022). A meta-analysis found that people experiencing homelessness in their 40s and 50s share similar frailty scores and geriatric conditions (e.g., functional and cognitive impairment, falls, incontinence, immobility) to stably housed adults aged in their 70s and 80s (Suh et al., 2022). Among people experiencing homelessness in England and Wales, the mean age of death whilst in homelessness is 43 years for females and 45 years for males, compared to 81 years for females and 76 years for males in the general population (Office of National Statistics, 2022). Thus, gerontology research among this population should not only focus on chronological age but also “accelerated aging” among people experiencing homelessness in their 40s and 50s.

Additionally, homelessness has been described as gendered with women experiencing “hidden homelessness” (Mayock and Bretherton, 2016). Research, practice, and policy have typically focused on male, visible representations of homelessness (i.e., in public spaces such as rough sleeping on the street, shelters, hostels), whereas women’s homelessness may be less visible due to reliance on accommodation via family and social networks (Mayock and Bretherton, 2016). Despite limited research, women experiencing homelessness require access to health-related information, services, and interventions tailored to their specific and multifaceted needs (Luchenski et al., 2018).

With migrant homelessness increasing in the UK and other countries (Baptista and Marlier, 2019; Consoli, 2023), it is important to be cognizant of the restrictions for migrant communities on access to housing and services due to legal, residency, or citizenship status (Mayock and Bretherton, 2016), in addition to language, literacy, cultural, and social norms (Richardson et al., 2022). Internationally, there is no formal legal definition of migrant, thus we use migrant and migrant status as umbrella terms regarding persons who change their place of usual residence, temporarily or permanently, irrespective of the reason for migration (International Organization for Migration [IOM], 2019).

Grounded in feminist epistemology (Ashton and McKenna, 2020) and the concept of epistemic injustice, the exclusion or undervaluing of knowledge due to assumptions about the credibility of the “knower” (Fricker, 2007), this study foregrounds narratives from older and middle-aged women experiencing homelessness, especially those from minoritized and/or migrant communities, who are often overlooked in research.

### Aim

We aimed to explore how technology, including digital health, can help or hinder older and middle-aged women to navigate paths through and out of homelessness.

## Research Design and Methods

### Study Design

**Figure 1.**
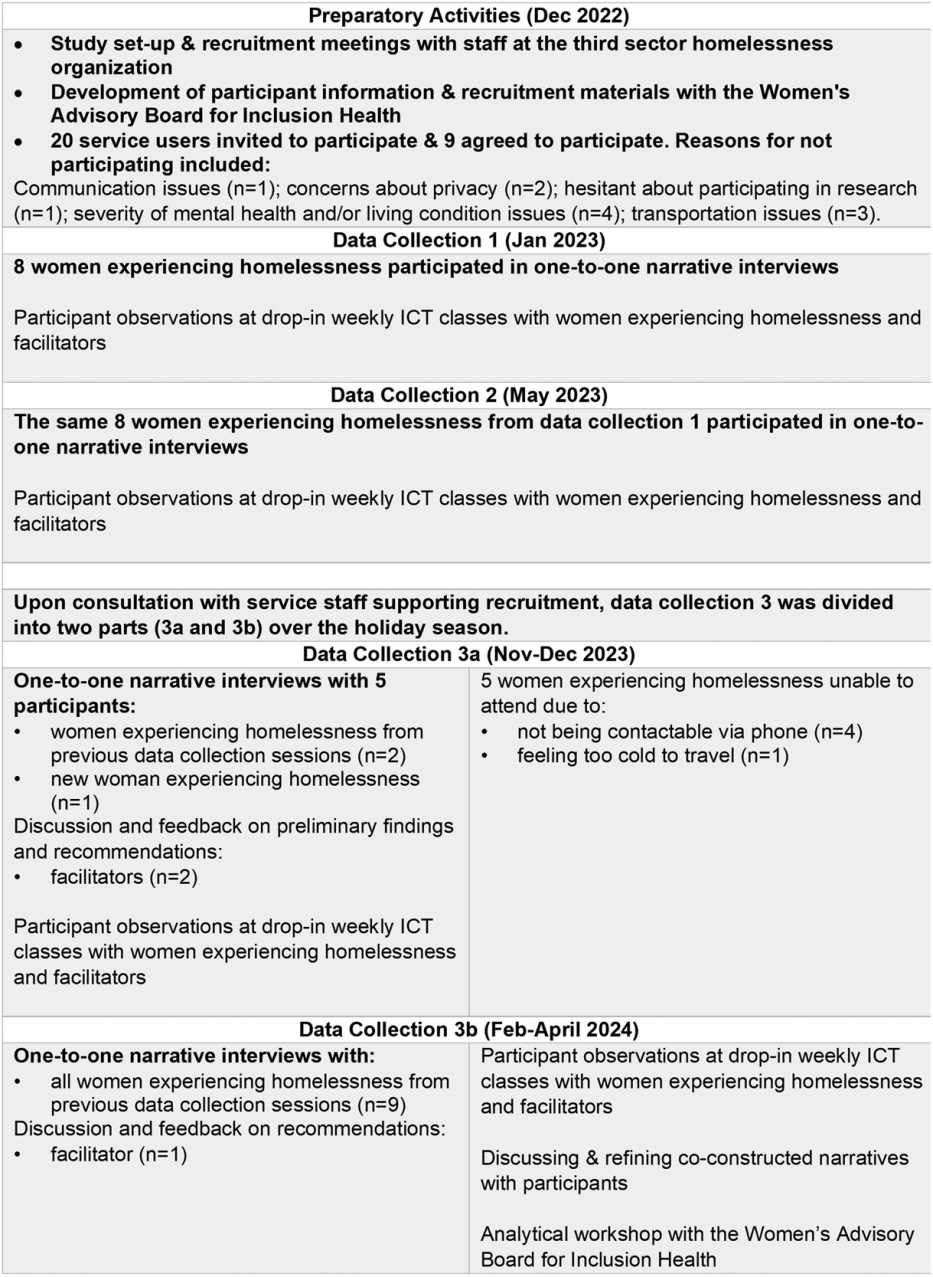
Overview of the study timeline and participants at each data collection. ICT = Information and communications technology.

Figure 1 presents an overview of the study’s timeline between December 2022 and April 2024. This qualitative longitudinal study utilized narrative interviews and participant observations, conducted at three time points over a 16-month period. The longitudinal design aligned with an interpretative approach to narrative as it enabled the participants and researchers to empirically test interpretations, following up on themes and questions in subsequent data collections (Andersen et al., 2017; Brown and Addington-Hall, 2008). Adherence to the COnsolidated criteria for REporting Qualitative research (COREQ) checklist was ensured (Supplementary File 3; Tong et al., 2007).

### Setting and Participants

#### Setting

Recruitment and data collection occurred at a third sector organization (i.e., registered charity) supporting women experiencing homelessness in London. The recruitment site’s dual structure, offering hostel accommodation with resettlement support and a drop-in center open 24/7 without requiring referrals, facilitated recruitment of women with diverse experiences of homelessness. Approximately 535 women accessed the drop-in center and over 100 were provided with accommodation between 2022 and 2023. At the time of this study, service users were aged between 18 and 75 years (mean age: 41 years), of which 57% were born outside the UK.

Data collection occurred at the drop-in center and some of the participants lived temporarily at the recruitment site accommodation. The drop-in center hosts free activities, including information and communications technology (ICT) classes, which were observed in this study. With permission from the ICT class facilitators, the researcher (SG) attended the two-hour weekly ICT classes held in the morning and afternoon. The number of participants varied due to the drop-in structure of the ICT classes which are held in a large room, with nine Microsoft PCs for service users and one for the facilitator. The classes aim to equip service users with ICT skills and to build confidence in using technology. Service users could also receive assistance with their own digital devices and refreshments were provided.

#### Participants

Together with the service staff supporting recruitment, we purposively sampled 20 women based on the study’s inclusion and exclusion criteria. We sought diversity across certain characteristics (e.g., types and durations of homelessness) which were discussed with the service staff supporting recruitment. Of the 20 invited women, nine agreed to participate. The reasons for not participating are shown in Figure 1. Eight women experiencing homelessness were initially recruited and an additional participant was included in data collections 3a and 3b to involve the perspective of a participant born in the UK. The number of participants was considered sufficient to meet the criteria of *information power*, which considers the relevance of participants’ information to the study aim (Malterud et al., 2016).

#### Women experiencing homelessness

This study used a broad definition of homelessness comprising rooflessness, houselessness, living in insecure housing, and living in inadequate housing (European Federation of National Organisations Working with the Homeless [FEANTSA], 2017). Inclusion criteria were women: aged 18 or over with lived experience of homelessness in England, able to communicate verbally in English, capable of giving informed consent to participate, and having access to everyday technologies (e.g., a phone, smartphone, or computer). Exclusion criteria included severe anxiety, distress, or violent and/or abusive behaviors. Despite the minimum age being 18, all recruited women were over 40, potentially mirroring a growing older population experiencing homelessness internationally (Baptista and Marlier, 2019). It is important to note that, despite being chronologically middle-aged, people experiencing homelessness often experience geriatric conditions (Suh et al., 2022). Staff from the recruitment site approached potential participants in-person, providing verbal and written information about the study, and asking whether they would be interested in meeting the researcher to discuss the study.

#### Facilitators of the ICT classes

Two female facilitators were invited to participate to provide insights into participants’ digital engagement and interactions during the ICT classes. They were approached in-person by the researcher and provided with written information about the study as well as time to consider their participation and discuss the study.

### Data Collection

#### Narrative interviews

The researcher performed in-person, one-to-one narrative interviews with participants in an on-site private room. Narrative interviews were audio recorded, lasting between 26 and 130 minutes (mean duration: 52 minutes), and transcribed verbatim by the researcher. The first interview focused on orientating participating women experiencing homelessness to the study, administering the demographic characteristics form to gather the self-reported demographic information presented in Table 1, and building rapport. Information about demographic characteristics was gathered to collectively contextualize the participants who co-constructed the composite narratives. To preserve confidentiality, potentially identifiable information about specific diagnoses were not elaborated in the findings but co-morbidities were embedded within the composite narratives. Aligned with narrative approaches (Josephsson and Alsaker, 2014), subsequent interviews aimed to elicit meaning-making between the participant and researcher regarding the study aim, using a broad topic guide comprising open-ended questions (e.g., “Please tell me about a few situations where you used technology to access help and care you need?,” “What happened before/during/after the situation?”) and prompts, which were not pilot tested (Supplementary File 1). Participants were compensated with a £10 grocery store voucher following each interview.

**Table 1.** Self-Reported Demographic Characteristics of Women Experiencing Homelessness at Baseline

Characteristic	Category	*n* (%) or mean (*SD*)
***N***		9
**Age**		55.9 (7.7)
**Ethnicity**	White-British	1 (11.1)
	White-Irish/Welsh	0 (0.0)
	White-other	3 (33.3)
	Asian-British	0 (0.0)
	Asian-other	0 (0.0)
	Black-British	1 (11.1)
	Black-other	2 (22.2)
	Other Ethnic Group^a^	2 (22.2)
**Country of birth by region**	Africa	3 (33.3)
	Asia (e.g., Middle East)	1 (11.1)
	Europe	3 (33.3)
	Latin America and the Caribbean	2 (22.2)
**Highest level of education**	No Formal Education	1 (11.1)
	Primary	1 (11.1)
	Secondary (e.g., O level, GCSE)	3 (33.3)
	University Degree	3 (33.3)
	Prefer not to say	1 (11.1)
**Employment status**	Unemployed/ Unable to find work	6 (66.7)
	Part-Time Employment	1 (11.1)
	Retired	1 (11.1)
	Prefer not to say	1 (11.1)
**Housing status**	Hostel	3 (33.3)
	Shelter (e.g., Night Shelter)	2 (22.2)
	Temporary Accommodation	3 (33.3)
	Living with Friends and/or Family	1 (11.1)
**Contact with family and/or relatives**	Yes	1 (11.1)
	No	6 (66.7)
	Prefer not to say	2 (22.2)
**Physical health issues** **(including comorbidities)**	Allergies	2 (22.2)
	Asthma	1 (11.1)
	Autism	2 (22.2)
	Chronic Pain	2 (22.2)
	Circulation Issues	1 (11.1)
	Sickle Cell Anemia	1 (11.1)
	Tuberculosis (in recovery)	1 (11.1)
	Prefer not to say	2 (22.2)
**Mental health issues**	Depression	1 (11.1)
	PTSD^b^	3 (33.3)
	Stress	1 (11.1)
	Other (e.g., visiting psychologist for unspecified reason)	1 (11.1)
	None reported	3 (33.3)

*Notes*: GCSE = General Certificate of Secondary Education; *SD* = standard deviation. Baseline corresponds to the participant’s first interview and data were self-reported by the participant using the demographic characteristics form. Eight of the nine women described experiences of sleeping rough but only one woman described currently sleeping rough when she was not able to access a night shelter.

^a^The Other Ethnic Group included one Hispanic or Latin American woman and one woman describing herself as from the Middle East.

^b^PTSD = post-traumatic stress disorder.

#### Participant Observations

Recognizing the interconnection between narrative and action (Josephsson and Alsaker, 2014), the researcher observed 10 ICT classes focusing on interactions between the participants and facilitators (e.g., conversations, nonverbal gestures, behaviors). Additional observations were integrated into the narrative interviews as participants demonstrated using digital health technologies and services (e.g., booking an appointment, accessing online health-related information). The researcher wrote field notes capturing descriptive and reflective elements directly after each observation (Atkinson and Hammersley, 1994).

### Data Analysis

Data collection and analysis followed an iterative approach inspired by Josephsson and Alsaker (2014) and earlier longitudinal narrative research (Andersen et al., 2017; Brown and Addington-Hall, 2008). Beginning early in data collection one, analysis was scaffolded by rich contextual descriptions in the researcher’s field journal and ongoing critical discussions between authors (SG, EM, and PR). The analysis process involved co-constructing composite narratives, presented as vignettes derived from, and incorporating, multiple participants’ data to capture specific aspects of the research findings based on the study’s aim (Johnston et al., 2023). The purpose was to identify coherent plots between and within participants’ data (Josephsson and Alsaker, 2014) through a cyclical process of reading, integrating transcripts and fields notes, and identifying meaning units or “narrative threads” that tie data around a single plot (Johnston et al., 2023). NVivo 12 software was used to manage the data. Possible plots and interpretations were tested in dialogue with the participants and wider research team. The outcome was three composite narrative vignettes, co-constructed from multiple participants’ data to capture individual experiences as well as overarching findings across participants (Johnston et al., 2023). All participants were deidentified and a single pseudonym was chosen by the participants and researcher to illustrate each narrative, while preserving confidentiality and incorporating participants’ “voices” via direct quotes and field note extracts (Johnston et al., 2023). Supplementary File 2 provides a worked example of how the composite narrative vignettes were co-constructed. Interpretations of the composite narrative vignettes using an intersectional theoretical lens are presented in the discussion section.

### Patient and Public Involvement and Engagement

The researchers met with the members of the Women’s Advisory Board for Inclusion Health (WAB) through workshops. The WAB contributed to the initial funding application, development of recruitment materials, analysis and interpretation of findings, and development of recommendations to promote the relevance and practical applicability of the study. The WAB was established in the Spring 2020 as an advisory board of women with lived experience of homelessness who work collaboratively with the research team as co-researchers. Since 2020, members of the WAB have met weekly for 2-hr workshops with the researchers and they are compensated on an hourly basis through temporary employment at the university, according to guidelines for public contribution and involvement (National Institute for Health Research [NIHR], 2021).

### Ethical Considerations

Participants provided written informed consent at the first interview and verbal informed consent thereafter. They were assured that participation was voluntary and that they could withdraw without consequences. Data were pseudonymized and stored securely at University College London (UCL). The study adhered to the Declaration of Helsinki (General Assembly of the World Medical Association, 2014) and obtained ethical approval from the UCL Research Ethics Committee [No. 23885/001]. Additional permission was granted from the Swedish Ethical Review Authority [No. 2023-00211-01] to share pseudonymized transcripts for analysis with the research team at Marie Cederschiöld University College in Stockholm, Sweden.

### Reflexivity, Positionality, and Trustworthiness

The study adopted an interpretative paradigm (Josephsson and Alsaker, 2014) and feminist epistemology (Ashton and McKenna, 2020), acknowledging the inherent influence of the all-female interdisciplinary research team. The researcher’s (PhD, Occupational Therapist) earlier work with women experiencing homelessness and research on digital equity informed the study’s context, but the research team had no prior relationship with the recruitment site. Building on earlier research (Brown and Addington-Hall, 2008), 3 reflexive strategies were used: questioning data, maintaining a field journal (SG), and consulting with a mentor (PR) and the WAB.

There are ongoing discussions on how the principles of trustworthiness can be applied to narrative research and we used guidance from earlier literature (Loh, 2013; McElhinney and Kennedy, 2022) to promote trustworthiness in the following ways. To promote credibility, we used a longitudinal study design which allowed for prolonged engagement and persistent observations, as well as ongoing opportunities for member checking with the participants themselves who also co-constructed and checked the narratives, including aspects of authenticity, before publication. However, this study is grounded in feminist epistemology which acknowledges that there is no singular representation of an authentic narrative. To enhance the study’s credibility and dependability, we incorporated triangulation of different methods (i.e., interviews and observations) and interdisciplinary perspectives (i.e., from the WAB and the research team including peer debriefing). To further increase dependability and confirmability, we archived the raw data and documented the data collection and analysis processes through an audit trail, in NVivo, and as shown in the worked example (Supplementary file 2). To assist the readers in making decisions about transferability, we ensured that the composite narratives included thick description and context. Moreover, the preliminary findings and recommendations were presented, in a PowerPoint presentation co-constructed with the participants, at an international conference on homelessness which provided an opportunity to test and refine the findings and recommendations with an audience of policymakers, clinicians, researchers, and people with lived experiences of homelessness.

## Findings

### Participant Characteristics

Figure 1 presents an overview of the study timeline and participants at each data collection. Table 1 presents baseline demographic characteristics of the women experiencing homelessness. The nine women, aged between 44 and 68 years (mean [standard deviation, *SD*]: 55.9 [7.7] years), reported various physical and mental health comorbidities, with only one woman in contact with her family and/or relatives. Eight of the women were born in countries outside of England (one woman preferred not to specify reporting it was in the Middle East).

### Narrative Findings

#### *Narrative one:* “*No, I’m not taking this telephone appointment.”*

Narrative one reveals the destabilizing impact of digital health on older and middle-aged women experiencing homelessness when they lack the ability to choose between in-person and digital health services. A one-size-fits-all approach to digital health overlooks diverse mental, cognitive, sensory, physical, and social needs, alongside issues of aging and literacy. Narrative one illustrates a desire for human connection and empathy, with digital health exacerbating mistrust and privacy concerns.

Juliana, a 57-year-old woman from Ecuador, has been engaging with homelessness services in London since arriving as a teenager with her boyfriend. Over the years, she has utilized various services, including those for victims of domestic violence and addiction treatment. Her life experiences and chronic health issues, including asthma, make her feel older than her years, exacerbated by time spent outdoors in cold, damp weather.

Recently, Juliana received a letter about a missed hospital appointment, but she is confused as she does not remember being informed about any such appointment. Due to her mobile phone being broken, she reluctantly resorts to using the homeless shelter’s shared phone to call the hospital, “If I have my phone, I wouldn’t call them with the phone in the shelter. Because, you know, it’s part of the things… privacy to me!.” At each attempt, Juliana is forwarded to an answering machine which is frustrating because she struggles with the speed of the automated recording.

Juliana grew up in an orphanage without the opportunity to attend school and struggles communicating in English which is not her first language. She is also living with autism and post-traumatic stress disorder (PTSD), so when she meets people, she tries to explain that her brain works a little differently, but it’s not always possible to do this when faced with technologies such as an answering machine. “I speak to, before the pandemic starts, I always go to them for face-to-face appointments,” she reminisces. Juliana wishes to speak to a real person, “No, I’m not taking this telephone appointment. Yeah, I refused them..” She’s tried using digital health services before, but it’s not always easy booking a GP appointment online when she doesn’t have a stable internet connection and she worries about the lack of privacy when talking about her health on the phone at the shelter in front of other people. Juliana decides to visit the hospital in person for clarification.

The hospital staff are busy, and they speak very quickly, she asks them to repeat themselves and she finally thinks she’s followed what they’ve said, except for one word, beginning with “N.” Juliana recalls that, “for two days it did my head in until the next day, in the morning, I phoned the nurse and I said, please tell me the name again.” Unable to get an answer, Juliana goes to the library to use the internet, but she realizes that she doesn’t know what she is looking for, where should she begin, the online auto-prompts for a word beginning with “N” are unhelpful. Juliana starts to feel helpless and that she has wasted her time and money to use the library computer, “[The nurse] she didn’t tell me how to spell it and I checked on the internet and they have different results.”

After a few moments, she remembers that the hospital staff mentioned that she would need to visit the pharmacist. She’s feeling tired but she makes her way to her local pharmacy as she’s familiar with the pharmacist, Mr. Patel. Juliana explains her situation and Mr. Patel suggests that it might be a Nebulizer. Juliana smiles as this is the correct word she was looking for. Mr. Patel explains about the Nebulizer and advises her to contact her GP, writing the word down on a piece of paper so she can show it if needed.

### Narrative Two: “Technology Doesn’t Judge You”

Narrative two highlights how technology, including digital health, can provide flexibility, autonomy, and anonymity for older and middle-aged women experiencing homelessness amid de-humanizing and stigmatizing in-person encounters. Creation and curation of online personas, through social media, gaming, and online learning offer escapism from feeling uncomfortable in one’s living space, body, or mind. However, reliance solely on technology may increase isolation, especially without in-person support networks.

Nadia, aged 48, does not always feel comfortable in her body or her surroundings. Sleeping on the street, she recalls waking up with strangers being too close to her or having her belongings stolen. For a long time, Nadia believes she is just an unlucky person due to her negative encounters with public services and authorities, including healthcare. “It’s like the universe doesn’t like me,” she tells herself. Originally from Iraq, Nadia feels that sometimes people misinterpret her communication style, especially the way she speaks passionately with hand gestures, as these could be perceived as being aggressive. Nadia is still acquainting herself with the etiquette and unwritten rules of interacting with public services and authorities in a new country.

Nadia feels less judged when using technology, “… even if you mispronounce the word, you wouldn’t feel embarrassed because the technology doesn’t judge you.” She can take her time to carefully construct text messages or emails and search for help online, “… the magic about the technology, you can actually change your words, you can delete it and you can’t do this with human being communication.” She prefers to book online or telephone healthcare appointments as she believes that people are less likely to judge her as a person, her accent, the way she looks, whether she had the opportunity to wash that day, or if she has changed her clothes. Despite Nadia’s transitory living situation, her smartphone provides a sense of home and consistency, she uses it to contact her family and services she needs, and it is where she stores her photos, contacts, and memories. Nadia thinks to herself, “Imagine, if you lose your phone, you go crazy! Because everything’s in here! Our photos, our memories, our appointment, our emails… address, phones, everything’s in there!.”

Nadia enjoys looking for inspiration about health and well-being on social media and playing video games, which she finds soothing and relaxing compared to her current living situation. She appreciates being able to construct and control an online persona, the person she hopes or wishes she could be in the future, “I can imagine that I am this guy in this moment. And then, I do what he is doing. It’s so nice when you see how he feels like. Or, if, if he’s clean or if he’s hungry, or if he needs to socialize..” But Nadia realizes that spending hours online can be isolating as she interacts with few people each day. Sometimes her “healthy” online persona feels very different to her everyday reality, because she does not have access to a kitchen or a fridge, and she is reliant on soup kitchens or donated sandwiches, which are not very nutritious.

### Narrative Three: “You Have To Be A Digital Person Now!”

Using interviews and participant observations from the ICT classes, narrative three focuses on learning new ICTs skills, or building on existing competencies, perceived as useful for an envisioned future through and out of homelessness. ICT classes provide a space for social connection and peer-to-peer learning. Technology is used as a memory aid and digital health equips the older and middle-aged women experiencing homelessness to become more informed and prepared for health care appointments, easing fears and uncertainties about memory issues, and motivating increased access to health care.

As Cecilia enters her weekly drop-in ICT class, she’s greeted warmly by the facilitator. Being in her 60s, Cecilia did not grow up using technology in Nigeria but feels eager to learn about it, so she doesn’t get left behind in a digital society. Cecilia thinks to herself, “But computer classes I like because I need to learn more. You have to be a digital person now!.” Last year, she was given an old laptop by a friend but wasn’t shown how to use it, so she has been keen to learn how to use it at the ICT class.

With practical support and emotional reassurance from the facilitator, Cecilia practices exercises such as writing emails, typing, creating spreadsheets, and searching for information online. She feels these skills could be very useful if she returns to work in the future and for managing her everyday life when she finds secure accommodation. Cecilia explains to the facilitator that she enjoys building on her existing skills, “I used to be a secretary, so I know how to do emails and stuff like that..” However, Cecilia continues that, “… just as I left emails were coming out. I mean I love emails. I don’t like texts. Because I get a lot of people when they contact me, they do text speak. In an email, you know like thanks, T-H-K-S. … That’s not for me. No, when you’re in your 50s or 60s you don’t need to do like the kids of 20..”

Sometimes the facilitator asks Cecilia to help the other women as she has become increasingly skilled at writing emails and searching for information online. Recently, she has been using the NHS website which includes videos about how to prepare for various health care appointments and what they will entail. Cecilia reads and listens along to the step-by-step video guides, taking notes on her smartphone. She has started to use her smartphone to keep a record of her health status, appointments she attends, and her medication and treatments. Preparing for health care appointments using videos and keeping notes on her smartphone works well for Cecilia as she has been experiencing memory issues lately. She used to feel embarrassed about not remembering, but she appreciated the facilitator’s explanation that computer storage is like a “human brain” that stores information. She values the way her laptop and smartphone can assist with her remembering things, “my brain is coming back!.” She continues to explain to the facilitator that, “I remember lots of things from my youth but just not the more recent things like this or that button.” Having made notes on her smartphone and searched for information on the NHS website, Cecilia feels more prepared for her upcoming appointment regarding her memory issues.

## Discussion and Implications

**Figure 2.**
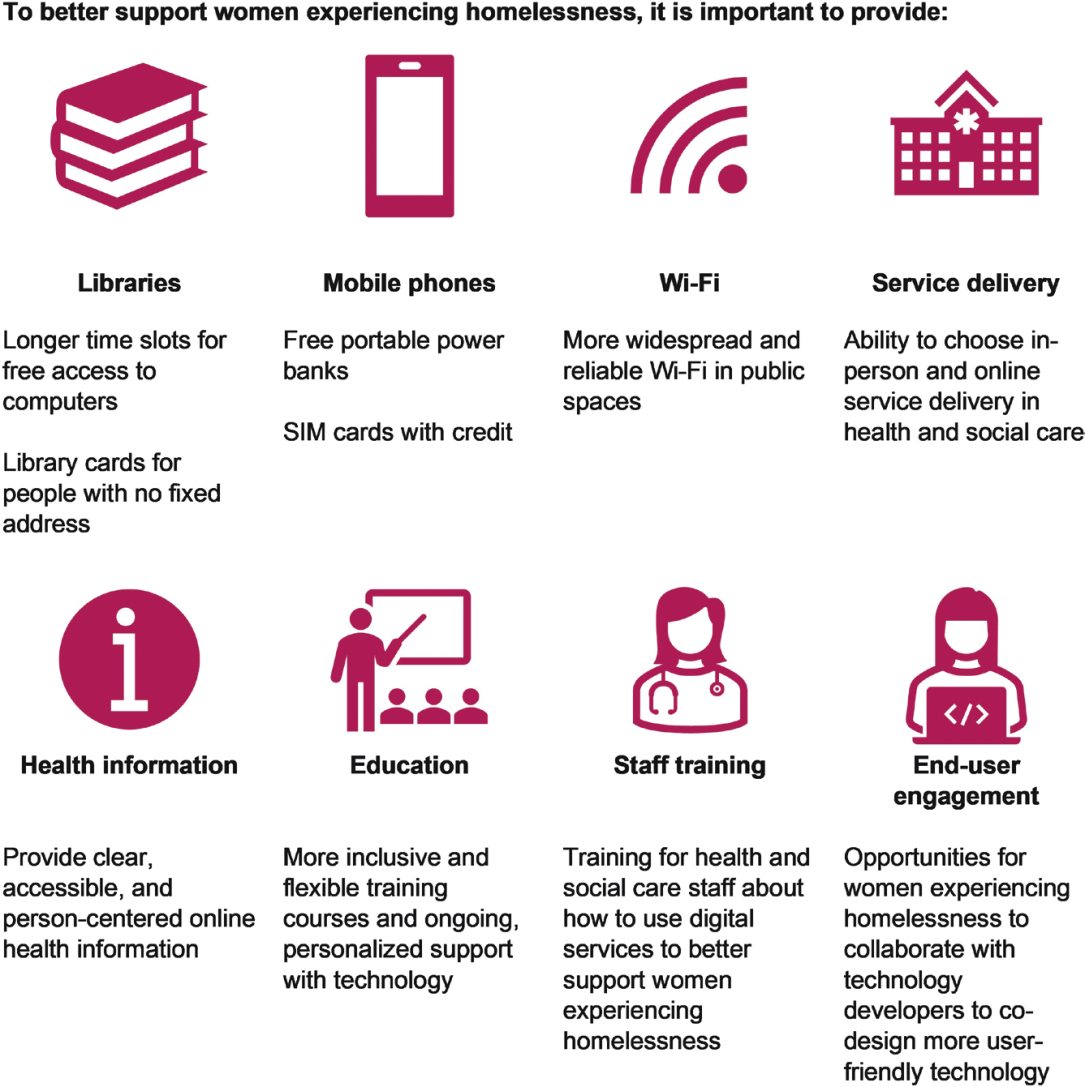
Digital health recommendations to better support women experiencing homelessness, co-constructed with women with lived experiences of homelessness.

The research team, participants, and WAB co-constructed recommendations based on the findings and literature (Figure 2). This study contributes to understanding digital health equity through an intersectional lens, which will be discussed in relation to the recommendations in this section. The openness of narrative methods enables various interpretations, and we acknowledge the contextual nature of our interpretations.

Our findings highlight how digital health may simultaneously exacerbate and ameliorate intersecting aspects of stigma and discrimination for the study participants. Narrative one illustrates how limited English proficiency exacerbates challenges using digital health services, necessitating in-person support to alleviate anxiety and to clarify technical health-related language. Consistent with earlier research (Higginbottom et al., 2019; Obionu et al., 2023), the women born outside the UK often encountered barriers to health service utilization, partly due to language limitations and lack of service awareness. Research suggests that women from minoritized and migrant communities may experience more negative attitudes from service providers compared to White British women (Obionu et al., 2023), and that cultural differences between themselves and health professionals may increase the risk of stereotyping, cultural insensitivity, stigmatization, and discrimination in health care encounters (Higginbottom et al., 2019; Obionu et al., 2023). Hence, *training for health and social care staff* should incorporate education on intersectional determinants of digital health equity. It is also recommended to provide *clear and accessible online health information* that caters to various literacy levels and language needs (Polillo et al., 2021; Richardson et al., 2022).

The findings, especially in narrative one, corroborate earlier research (Grossman et al., 2022) on the role of public libraries in helping migrants to adjust to life in a new country. Libraries provide access to technology, resources and education programs (e.g., ICT skills), which may enhance confidence in skill-building and socializing. To leverage *libraries as community resources*, it is recommended to extend free computer access and to ensure library card access without a fixed address (Dowdell and Liew, 2019). However, library services and resources may not currently be tailored to the needs of older and middle-aged women experiencing homelessness. Research indicates that reliance on public libraries for internet access may increase digital inequities and exacerbate privacy and data security concerns (Rhinesmith, 2023), creating barriers to digital health for marginalized populations (Rabet et al., 2024). Due to the transient nature of homelessness, *widespread and reliable Wi-Fi* in public spaces, such as mobile Wi-Fi hotspots, potentially connected to a person’s library card for additional security (Rhinesmith, 2023), are recommended. Moreover, participants and the WAB recommended providing *free portable power banks and SIM cards with credit* to women experiencing homelessness to alleviate reliance on shared, public phones, thus preserving privacy. Aligned with the literature (Heaslip, Green, et al., 2021; Heaslip, Richer, et al., 2021), they acknowledged the risk of these items being lost, stolen, or sold for drugs.

Our findings challenge the conventional view of technology and digital health users as young, male, and with a higher education and socioeconomic status (Figueroa et al., 2021). Recently scholars have problematized ageism among older people experiencing homelessness (Weldrick and Canham, 2024), as well as digital technology-based ageism which views older adults as passive and dependent on others (e.g., caregivers or health and social care professionals) to use digital technology due to their assumed frailty (Mannheim and Köttl, 2024). A scoping review (Ekoh et al., 2023) found that digital technologies are important for older migrants’ health and well-being, to maintain and develop social support networks, to develop coping strategies for migration-induced stress, and preserve connections to their culture if desired. Narrative three reveals how education in ICT skills may help older and middle-aged women experiencing homelessness from minoritized or migrant communities to access online information and support regarding memory issues. Earlier research (Ma and Joshi, 2021) suggests that cultural beliefs may contribute to the stigma of memory issues and/or dementia among migrated older adults in the UK. It is recommended to provide inclusive and flexible *ICT courses and digital health education*, recognizing that digital health information and diagnoses may be potentially stigmatizing depending on cultural and social norms (Ma and Joshi, 2021).

Narrative two revealed cultural and gendered dimensions of stigmatizing and discriminatory attitudes encountered through in-person health care, which have been linked to reduced caring behaviors perceived by women experiencing homelessness (Gaber et al., 2022), and unmet health care needs among female migrants (Nyikavaranda et al., 2023). In narratives two and three, technology helped the women adapt to social and cultural norms of service delivery in a new country. Despite an overall preference for in-person communication, some women, especially the middle-aged women experiencing homelessness, found online service delivery more flexible and less stigmatizing. Based on our findings and earlier literature, it is recommended to *provide choice between in-person and online service delivery* to promote agency and reduce reluctance to engage with services, due to suspicion of asking for help from strangers, fear of being detained or deported, and insufficient sensitivity regarding health (Nyikavaranda et al., 2023).

Corroborating earlier research, mobile and smartphones were described as practical tools for storing and searching for information, maintaining contacts, and engaging with essential services, including digital health services (Heaslip, Richer, et al., 2021; Raven et al., 2018). As illustrated in narrative two, mobile and smartphones also held emotional and symbolic value, serving as a “home” for cherished photographs, social connections, and memories. To better understand perceived meanings of digital technology and digital health for marginalized populations, it is recommended to involve them through *end-user engagement* in designing and delivering digital health, thus helping to mitigate challenges around privacy concerns and enhance self-efficacy (Coetzer et al., 2024; Wilson et al., 2021). Our findings will inform future collaborative and participatory research, through workshops incorporating narratives and other methodologies, to co-design a digital health intervention *by* and *for* women experiencing homelessness.

### Strengths and Limitations

The composite narrative vignettes, co-constructed by the participants and researcher, contribute to the knowledge base on qualitative longitudinal research among women experiencing homelessness. Involving women experiencing homelessness, enhanced the study’s rigor in interpreting findings and developing practice and policy implications.

The longitudinal design enabled the researcher to spend time in the participants’ environment, meeting service users, and attending activities such as the ICT class, thus normalizing her presence for the participant observations (Atkinson and Hammersley, 1994).

Purposive sampling was used to enhance the rigor and trustworthiness of the findings by aligning the participant group with the study’s aim and eligibility criteria. This study’s findings, derived from a relatively small sample, do not claim generalizability, or an exhaustive explanation of all potential experiences of digital health among women experiencing homelessness. However, the meaning-making process of narrative research is suited to a smaller sample followed over a longer period (Malterud et al., 2016). Due to the narrative approach and limited number of participants, the present study avoided drawing comparisons between women born outside the UK and the woman born in the UK; however, this topic may be explored in future research using different methodologies and with more data.

## Supplementary Material

gnaf021_suppl_Supplementary_Material

## Data Availability

The data that support the findings of this study are available from the corresponding author upon reasonable request. This study was not preregistered.
