# Supplementary material for: An Intersectional Perspective on Digital Health: Longitudinal Narratives and Observations With Older and Middle-Aged Women Experiencing Homelessness
Source: Gerontologist. 2025 Jan 27;65(4):gnaf021. doi: 10.1093/geront/gnaf021 (PMC11973565; doi:10.1093/geront/gnaf021)
Supplement: gnaf021_suppl_Supplementary_Material [file gnaf021_suppl_supplementary_material.docx]

**Supplementary Material**

**Supplementary File 1.** Interview and observation guide

**Study title:** Pathways And Technology for women with experiences of Homelessness: A qualitative interview study (PATH-study)

**Overview and prompts for narrative interviews and participant observations**

Following a narrative interview approach, the researcher will not set out with standardized questions. For each interview/observation session, the researcher will be inspired by the four basic phases of a narrative interview (Anderson & Kirkpatrick, 2016). The focus is on the participant’s (the woman experiencing homelessness’) account, she will choose what to say/not say, controlling the content and pace of the interviews and observations.

1. **Introduction and explanations about the research**

This phase focuses on developing rapport with the participant and orienting them to the research.

The researcher will explain about the interview and observation process (e.g., that the session will be audio recorded and will last approximately one hour), and the purpose (e.g., the researcher would like to hear the participant’s experiences of accessing and using technology in their own words).

The researcher will take informed consent and during the first interview, demographic information will be collected using the demographic characteristics form.

1. **The narrative**

The researcher will begin with an open-ended question (e.g., “Please tell me about a few situations where you managed to use technology to access the help and care you need, and the opposite where you had difficulties, or it did not go well.”).

The intention of this phase is for the participant to begin telling their story about experiences of how technology, including digital health, can affect their everyday lives, such as accessing the help and care they need.

The researcher will use non-verbal encouragement (e.g., facial expressions, hand gestures) to encourage the participant to talk freely.

The researcher will avoid interrupting until there are clear signs that the participant has finished telling the story (to respect the flow of the participant’s story and to minimize short, factual answers).

The researcher will pay close attention to narrative material based on the participant’s stories/plots (e.g., “first I did this, and then I thought this, and I went to this place to ask for help… and she told me about this…”).

1. **Questioning phase (in combination with participant observations)**

This phase involves active listening by the researcher as they use the participant’s own language to fill in any gaps in the narrative.

The researcher may ask for more detail about an issue of interest (e.g., “What happened then/before/after…?”, or “Can you say a little more about…?”).

The researcher will avoid asking for opinions/ attitudes or asking why questions.

Instead, the researcher will focus on “how” questions related to the participant’s experiences.

The observation of an activity involving technology will be embedded in the interview, it may occur at any time depending on the preferences of the participant, and the participant will choose the type/ form of activity (e.g., making an online appointment or using an app on a smartphone) (Josephsson & Alsaker, 2014).

In the questioning phase, the researcher will use prompts to elicit richer narrative material during the observations (e.g., “please show me how you did that…”).

1. **Conclusion**

Finally, the researcher will conclude the interview/ observations and will check if the participant has any other questions.

The researcher will explain the next steps (e.g., transcribing of the interview and whether the participant would like to read/ provide input on the transcript, arranging the next meeting).

**Guiding notes:**

Location of interviews/ observations: at the UCL offices, third sector homelessness organization, or another location chosen by the participant.

Field notes: the interviews will be audio recorded; however, to minimize the observer effect, field notes will be taken directly after the interviews/ observations.

Analysis: an iterative (ongoing) approach will be used to analyze the data, this includes providing opportunities for participants to read and give input on transcripts from each interview which may then inform the subsequent interview.

At all times, the researcher will use a flexible, respectful, and person-centered approach based on the preferences/ needs of the participant as well as the expertise of staff and management at the recruiting third sector homelessness organization who are familiar with the participants.

**Supplementary File 2.** Worked example of co-constructing a composite narrative (narrative three)

**Draft 1 of the narrative**

The data were first organized into a long list of quotes from interviews and field note extracts from the observations that pointed to the properties and dimensions of the main finding (Johnston et al., 2023). Through reading and re-reading the quotes and field note extracts, a “narrative thread” (i.e., *ICT classes as an empowering pathway through and out of homelessness*), tying together the composite data around a single narrative plot, was chosen (Johnston et al., 2023). The quotes and field note extracts were rearranged to capture the participant’s descriptions of the “narrative thread”. Supplementary Figure 1 presents an extract of this process.

| **Identifying quotes based on data coded to *ICT classes as an empowering pathway through and out of homelessness***  *P: But computer classes I like because I need to learn more. You have to be a digital person now!*  *I: Yeah?*  *P: Because this is the now, er… er…. umm… er …*  *I: The way society is going more and more?*  *P: And I need to know so much more… because it is the reality! We are digital!*  *I: Mmm, absolutely.*  *P: Er.. era… era?*  *I: Yes, that’s it.*  *P: Era, digital era.*  *[P07 Data collection 01, interview]*  **Constructing the narrative thread**  Cecilia likes the computer class and is eager to learn about technology, so she doesn’t get left behind in a digital society. |
| --- |

**Supplementary Figure 1.** Constructing the narrative thread

**Draft 2 of the narrative**

Building directly on the participant’s quotes and language, minor changes were made to enhance readability and narrative flow (Johnston et al., 2023). Supplementary Figure 2 presents an extract of the process of identifying relevant quotes and developing an initial composite narrative, using different participants’ descriptions and experiences related to the “narrative thread” from Supplementary Figure 1. Data from all data collection timepoints were incorporated into this narrative, including 19 interview quotes from eight participants (women experiencing homelessness n=6, ICT class facilitators n=2) and 13 field note extracts from observations at six ICT classes (bolded and italicized below). Examples of quotes used verbatim in subsequent drafts of the worked example are bolded.

| **Identifying additional quotes based on data coded to *ICT classes as an empowering pathway through and out of homelessness* and constructing the narrative thread in the form of a composite narrative** | |
| --- | --- |
| **Description/Unit of meaning** | **Quote and/or field note extract** |
| Greeted warmly by facilitator | Once she had warmly greeted the different people in the class, she sat down and placed her belongings down. Participant 06 spent several minutes tinkering around with the PC, moving the mouse around whilst she surveyed the room.  [***Observation of ICT class 03, Data collection 02***] |
| Likes ICT class – same time/place and drop-in | Participant (P): But to be honest with you, it’s about just creating a space where they come from, consistently come and do this because without consistency or regular access to IT.  Interviewer (I): And coming as they are, so do they need to bring anything?  P: Totally!  I: And having, are there set days? Does it change?  P: No, it’s Thursday.  I: And is it two classes? In the morning and afternoon?  P: Yes, yes.  I: And maybe having a set time.  P: Yes. Yeah, that's it. And then if not, then they've got access elsewhere during the week to be able to practice as well.  [P10F Data collection 03a, interview]  -----------------------------------------------------  P: Yeah, there’d be no reason why we’d turn the women away from the classes. Though the IT class, the ESOL class, sewing in a class called Life Skills are all funded. So, we do have to get enrolment forms from people. Because we're supposed to have a certain fulfilment of numbers across the year and want to get the funding for the following year, but we make it very clear if someone doesn't want to fill out one of those enrolment forms, they must still be allowed access to the class. It's not a prerequisite, yeah.  [P11F Data collection 03a, interview] |
| Did not grow up using technology but eager to learn to avoid being left behind in a digital society | P: **But computer classes I like because I need to learn more. You have to be a digital person now!**  I: Yeah?  P: Because this is the now, er… er…. umm… er …  I: The way society is going more and more?  P: And I need to know so much more… because it is the reality! We are digital!  I: Mmm, absolutely.  P: Er.. era… era?  I: Yes, that’s it.  P: Era, digital era.  [P07 Data collection 01, interview]  ----------------------------------------------------  P: You know, we live in the world that is all on the internet, all communication, all the world. And the telephone appointment.  I: Yeah?  P: Is that the word…?  I: Umm, doctors and…?  P: Yeah, all is digi, digital!  I: Digital, yeah.  P: Digital culture.  [P07, Data collection 02, interview] |
| Given an old laptop but not shown how to use it | P: Yes, it’s nice (laughter). My daughter want me to get his. So ummm… so ummm. I get this from [anonymized homelessness charity]…  I: …Ok so [anonymized homelessness charity] give you a laptop and smartphone and do they show you how to use it?  P: I go to class there. Tomorrow, I’m supposed to go to class. But I can’t go, I have another meeting to go to.  [P01 Data collection 01, interview]  -----------------------------------------------------  P: Mmm, yeah. This is the phone.  I: Ok. And did you get that from [anonymized homelessness charity]? Was it…  P: Yes, you remember I told you… I did, yeah. So… [showing the phone to the interviewer].  I: Because this is a smartphone now. Isn’t it?  P: I don’t know. This is not from [anonymized homelessness charity]. This is from my [other anonymized homelessness charity] people.  I: Ah ok.  P: They gave me. Yeah, a couple, maybe a week or 2. Maybe it’s a week now. Or 2 weeks...  P: … He just sit there and he do it.  I: Ok, so like your old numbers and…  P: Everything.  I: … he transferred it.  P: Everything, in this one.  I: And he helped you with that?  P: Mmm, but don’t ask me… I don’t get how to do it.  (Both laughing together)  P: I go home the night, the evening, and I said, “Me God, what me know about this phone?”.  I: Yeah?  P: It’s new and he told me about how to do all the things (laughing). But whenever I feel, I feel that I’m not doing it!  I: Why do you feel that?  P: Because of how he do it all. And when I do it, and I know he told me to do it.  I: Yeah?  P: But, God, I can’t remember these things.  I: Mmm, mmm.  P: And he then, the next day I call him. And he said, “[anonymized participant’s name], come, come back… come back, let’s see.”. I said, “No, I’m not coming back. I’m a big girl. I’m not supposed to.”.  [P01 Data collection 02, interview] |
| Eager to learn and start using own laptop in the ICT classes | For approximately 15 more minutes, Participant 01 was the only service user still in the class and she was visibly happy when she successfully completed a task. Once she felt that she’d finished the task, she looked over to me and exclaimed, **“my brain is coming back!”. She continued to explain, “I remember lots of things from my youth but just not the more recent things like this or that button.”** She explained that she had two certificates in computer skills but that she forgot things and “blocked” them out after her belongings were stolen. “Unblock, unblock” she said for emphasis.  [***Observation of ICT class 03, Data collection 02***]  ----------------------------------------------------  Participant 01 explained that she wants to practice more and that she has a laptop, but it is in storage, and she doesn’t want to carry lots of things. I observed that the facilitator was very patient with the participant, and she suggested that the participant could come to the computer room and practice any day. The participant smiled, gathering up her belongings to leave and said, “I’m glad I came”.  [***Observation of ICT class 03, Data collection 02***]  ----------------------------------------------------  Participant 01 explained that she planned to get her laptop out of storage as she thought it was now time to start using it again. I wondered if seeing the other women using laptops had inspired her as she’d previously mentioned having a laptop in storage but had expressed some hesitancy about having to carry it around. The facilitator was encouraging about this plan and suggested that it could be helpful to practice on the technology that she would actually be using on a daily basis.  [***Observation of ICT class 05, Data collection 02***] |
| Practices exercises in class (e.g., writing emails, typing, creating spreadsheets, and searching for information online) | Simultaneously within the room, it ranges from women working on basic tasks (e.g., logging on and off, or creating an email account, exploring an internet search engine, planning a travel route, checking details of a service etc.), to pre-defined tasks with different levels of complexity (e.g., typing up a word document with formatting exercises, writing a shopping list, or accounting/budgeting on Excel). Consistently, through the classes I’ve noticed that the facilitator tries to remind the attendees about the need to log out at the end of the class (i.e., emails accounts and the PC itself) for security reasons.  [***Observation of ICT class 04, Data collection 02***] |
| Feels ICT skills could be useful in the future (e.g., if she returns to work and for managing her everyday life when she finds secure accommodation) and she used to be a secretary using email but not text talk which she feels is for the kids | P: You know, I was thinking if I do, er, get a proper Visa and things and then when I’m working I will have to use technology to do…  I; Yeah.  P: to make doctor’s appointments… to make NHS applications.  I: Exactly.  P: I think I could do it.  I: Yes, I was going to say, how do you feel about that? Do you think you’ll maybe, I don’t know because they also do courses here where they help… do you think you’d like that maybe?  P: Yes. They’re probably quite useful but I haven’t been to any IT class yet.  [P05 Data collection 01, interview]  -----------------------------------------------------  P: **I used to be a secretary, so I know how to do emails and stuff like that.**  I: Yeah, well that would be interesting to speak with you about then, yes.  P: Because I basically **just as I left emails were coming out. I mean I love emails. I don’t like texts. Because I get a lot of people when they contact me, they do text speak. In an email, you know like thanks, T-H-K-S.**  I: Oh, like abbreviations. Or?  P: Yeah. **That’s not for me. No, when you’re in your 50s or 60s you don’t need to do like the kids of 20.**    [P09 Data collection 03a, interview] |
| Facilitator can help if she makes any mistakes, if she forgets how to do something, and because the facilitator encourages her to feel more confident | The facilitator gave her a fist pump and we all laughed. She required some help from the facilitator to email the document to the facilitator and to be reminded to log out. She stood up and said, “The best I ever do!”, clearly satisfied with her achievements from the ICT class today.  [***Observation of ICT class 03, Data collection 02***]  -------------------------------------------------------  After a few moments, she called out to the facilitator, “Help, help… please!”. The facilitator rushed over, and I anticipated that the facilitator expected it to be something urgent, but Participant 06 explained that she needed help to find a website to practice her typing. She spoke in a determined tone about wanting to develop her typing skills. The facilitator signposted her to the relevant website and then went to help another woman in the class.  [***Observation of ICT class 05, Data collection 02***] |
| Reminisces about being a student back home | P: Because… the teacher teaches us at school back home and you will do something on the board, just showing us something what to do (gesturing as if writing on a (chalk)board).  I: Yeah, like that…  (Laughter)  P: Ah… then he do it, as if for maths, English, for anything. And then he starts to give us a… straight away after.  I: Yes.  P: Do this!  I: So, you have to copy? And how it’s done?  P: No, I can copy it. But I don’t I just watch his, how he’s writing… (mumbling) … just writing.  I: Yes.  P: And I answer every question without writing!  [P01 Data collection 02, interview] |
| Helps her peers in the ICT class | P: Support, yeah, yes and no. And I think that's something to use a bit more as well for those that are confident, just that will help somebody else out. But, umm, yeah, I think you’ll hear me saying, I don’t know, I don’t know (laughing).  I: Is that deliberate?  P: That’s so deliberate!  [P10F Data collection 03a, interview]  -----------------------------------------------------  The facilitator was busy helping another woman, so she asked a service user to check if a fellow service user was ok and what she was doing. They spoke for a little while. Participant 06 continued to look at the start screen and slowly moved her mouse around, hovering over the different applications in the start menu. It appeared that the women had helped each other to progress with the ICT exercises.  [***Observation of ICT class 07, Data collection 03***] |
| Uses the NHS website and the step-by-step online guides to prepare for healthcare appointments | P: Er, er, I think it’s lymph, Lymphoedema.  I: Yeah, Lymphoedema.  P: Many word, yeah. Umm, about the stockings. How to wear them? Step-by-step. You know, where do they order? The NHS does provide videos on that.  I: Yeah. Did they for example, you know with the, er, PAP, smear test, did they signpost you, the nurses, the doctors, to watch those videos? Or did you have to find them yourself?  P: I did find them myself.  I: Mmm.  P: Er, through the lack of knowledge.  I: Hmm.  P: Umm, the GP… umm, did first prescribe… umm, you know, did a prescription. Er, however, when I have the other appointment at the hospital for the same reason. Then they, er, then they also suggest we are going to er… but I took the initiative to search. Partly, to have more information.  I: Mmm.  P: Right?  I: Because it sounds like a great resource but I’m not sure if everyone knows about it even. You know…  P: It depends how interested are you.  I: Mmm, yeah?  P: Or if you know already.  I: Yeah, maybe.  P: But I don’t.  I: No, no.  P: So, I did search as much as I can!  I: Mmm, mmm.  P: Until I’m satisfied and relieved! (Pulls a humorous face)  [P02 Data collection 02, interview] |
| Reads and listens along to the video, taking notes on her smartphone | The facilitator advised the woman to take her time and read the information because “sometimes we are too quick to click and it can create problems later.”  [***Observation of ICT class 06, Data collection 03***]  -----------------------------------------------------  P: I think videos are the way to go in terms of information, yeah. So that is just all with [Participant 01’s anonymized name] audio as well as reading, especially when language is something. So, it's something that can be done slowly so that they can hear it and try it and understand a bit more of what they need to do. You know, umm. One of the things like, it’s just about accessibility. What I need to do for, and I have to remember this for like [Participant 01’s anonymized name] the screens, the text is too small for her.  I: Ok.  P: And she’s got arthritis in her hands so she gets tir…  I: Does she get tired after a while?  P: That’s it, yeah. So, what can we do or put things in place? So, I can imagine that's going to be even more difficult using the phone.  [P10F Data collection 03a, interview]  -----------------------------------------------------  The facilitator suggested that she click the sound button and a voiceover started to read aloud the instructions. Participant 06 exclaimed, “Wow!”. Then, Participant 06 moved to the next page and continued to use the voiceover to help read aloud the text and to pause and take notes on her smartphone.  [***Observation of ICT class 07, Data collection 03***] |
| Uses her smartphone (and email) to (practice and) keep a record | After the short break, the co-facilitator suggested that Participant 01 focus on working with navigating/planning her journey on the smartphone since this is the technology that she will actually be using after the class. The co-facilitator prompted the participant to open her City Mapper app. Initially, the participant said she did not know what that was and did not have it but when the co-facilitator gestured to it on the participant’s smartphone screen, the participant nodded and opened the app…. After exploring the app for a little time, the participant asked how the app would know where she is and where she is going. The co-facilitator explained that the smartphone had GPS and when it had asked for permission to track the location this meant that it would use a satellite to walk along with you. The participant stamped her foot on the floor and exclaimed “Technology, wow!”. She continued to giggle and look at me. After practicing using the app on the smartphone, the participant confidently stated, “I know this route!”.  [***Observation of ICT class 01, Data collection 01***]  -----------------------------------------------------  P: Oh yeah, because I just had sort of everything in there. It was like my diary was in there. It was everything, everything I was doing. Yeah. What's happened to me in the past? Cause I I log everything…  P: Yeah (laughter). No. it’s just in case I forget things.  I: Yeah. Do you think it helps your memory?  P: Yeah, because my mum, my mum had dementia and Alzheimer's. She had rheumatoid arthritis, breast cancer and bowel cancer. So, I'm petrified because one of my friends, that's not my friend now and I've known her for 50 years. She said, [Participant 09’s anonymized name], if you get to mention, does it matter who's gonna know, you’re on your own. And one of the clubs I go to, the woman said to me, does she come here [Participant 09’s anonymized name]? I said, no she lives in [anonymized location]. She said that's a disgusting thing to say, but it's something that I can't forget it's at the back of my memory.  [P09 Data collection 03a, interview]  -----------------------------------------------------  P: E-mail, e-mail, e-mail is is is the way I think is first because you have a record of the communication. And if you are in the phone, you always get a different person. You don't speak to the same person most of the time, and I think the communication gets lost on the phone because I know they said they record everything. But I think it's much easier if you want to go back and look at the e-mail so it’s all there.  [P03 Data collection 03a, interview] |
| Keeping notes to help her remember information | P: So, I was… uh God! (Background sound of the participant zipping bag). And it hurts me so much.  I: Really?  P: Yeah.  I: In what way?  P: … because (pause). I passed my (laughing to herself) my computer course. Two certificate. And sometimes, now I know I can’t remember this… see even tell me something…  I: Mmm  P: … and I don’t remember… just er, you know.  I: Mmm  P: So, generally I tell her I want to write down the things.  I: Ok.  P: If I write it down and I go home, then I will you know…  I: Read it?  P: Yes, and see if I can…  I: Take your time?  P: Yes. So. It’s last week now, she said that we will try and write down things and such.  I: Yeah.  P: I was a computer brain!  [P01 Data collection 02, interview] |
| Facilitator compares computer storage to the human brain, remembering things from youth but not more recent things | The instructions said that the PC had more “memory” than a laptop, Participant 06 said, “Wow” and asked if this was true. The facilitator explained that this was correct and used the metaphor of the hard drive being **like a “human brain” that stores information.**  [***Observation of ICT class 07, Data collection 03***] |
| Likes the security of using Google drive/the Cloud to store and save digital memories even if she loses her laptop or smartphone | I: So, you go onto Google Drive?  P: Yes. So that they can get the free account on Google Drive and still be able to use the clouds.  I: For Word? And is it the program, Sheets?  P: Yeah. Yeah, all of those. And they still can then save the work. So, it's not that they come and use the computer, then it just disappears. So, they've got a history, they've got a trail and then that also helps trigger me, reminds me of what they were last working on.  [P10F Data collection 03a, interview]  -----------------------------------------------------  Participant 01 spent some time moving her mouse around the screen, seemingly curious about the effects of hovering over different icons, for example hovering the mouse over the profile and the “add your picture here” option would temporarily appear. She seemed to find the temporary appearance and disappearance of options fascinating and a little confusing. After a few minutes, the facilitator came over and suggested that the participant check her (Google) Drive to see what she had been working on from the previous class. Using an inquisitive tone, she asked the facilitator about the previous week’s class. It was a little unclear whether she had forgotten the previous week’s work or wanted the facilitator to remind her of her achievements from the previous week. The facilitator gave a brief summary of the previous week’s work and helped the participant to scroll down and locate the correct document.  [***Observation of ICT class 03, Data collection 02***] |
| Having made notes on her smartphone and searched for information on the NHS website, feels more prepared for her upcoming appointment with her GP regarding her memory issues | P: Actually, I just received a text message today and I'm going for an operation in March and when I went to the [anonymized hospital name] website, I really like the way, let me show you [Participant 03 showed the website on her smartphone]. It’s beautiful!  I: Oh really, do you think it’s well done?  P: Yeah, I really loved it! The way they make it very clear, very simple! And for the first time in my life, I actually, they umm, yeah images [Participant 03 navigates to the image section on the website]. Yeah, look at that! They send me saying, what you should expect.  I: Ok.  P: And they send me a photo of the team so I can see.  I: Yeah, so hopefully you can recognize.  P: Yeah, and they give their names as well. And also look at that about how you can arrive, how you prepare.  I: To prepare.  P: And if you want to have someone with you. And if you don’t want to, you can actually, I didn’t know about that, there is actually they can provide someone from the staff to be with you as a companion. And if you’re running late. Yeah, they may not be able to see you but you will. And also at the beginning they said you might have to wait for er 30 minutes, maybe.  I: Yeah.  P: And also, who you will see, umm, all of these procedures, privacy policy. They actually mention that the NHS has a new, er, free WI-FI. Which is actually a very good move for people who don’t have, I have it already but if there are some people they don’t have. But that means it’s good. What will happen after your appointment. I loved it!  [P03 Data collection 03b, interview]  -----------------------------------------------------  P: And other people, I do things for them, but I can't from like, from my things happen to me, I can't remember.  I: How is your memory at the moment? Cause I know you said sometimes even in the computer class, sometimes things will come back to you.  P: Yes, yes.  I: But other times, did you say there were issues with your memory?  P: Yeah. Because what I was saying to her, put the thing on a paper.  I: Write it down?  P: Write it down. Let me study it because you know they way we study because that is what I do when, when I go to computer school.  [P01 Data collection 03a, interview]  -----------------------------------------------------  P: Well, I want to just revise, revise, revising.  I: Revise it?  P: Yeah, but it can’t stay, it can’t stay in my brain.  I: Have you seen a doctor about your memory or anything like that?  P: Mmm.  I: Do you think you would, maybe?  P: I saw it, I saw something on my phone. This man who do memory thing.  I: Yeah?  P: And I want to do it, I want to but I don’t have the money to pay.  I: Maybe you could speak to your GP about it. Your doctor maybe to see about the memory issues.  P: Yeah, I’m going to see the GP.  [P01 Data collection 03a, interview] |

**Supplementary Figure 2.** Constructing the narrative thread by incorporating other participants’ descriptions and experiences

**Draft 3 of the narrative**

The narrative was further developed through discussions with the research team, including developing a summary of the narrative and identifying contrasting examples that communicate some of the challenges encountered in relation to the *ICT classes as an empowering pathway through and out of homelessness*. Revisions are bolded and underlined. The version shown in Supplementary Figure 3 was presented and discussed with the participants during data collection 3b.

| **Constructing the narrative thread in a composite narrative, including contrasting examples**  Summary  Drawing on participant observations from the ICT classes, this narrative plot is about actively learning new ICTs skills, or building on existing competencies, perceived as useful for an envisioned future through and out of homelessness. The ICT class provides a space for education, problem-solving, skill building as well as social connection and peer-to-peer learning. **Technology is used as a memory aid and digital health equips the older and middle-aged women experiencing homelessness to become more informed and prepared for forthcoming healthcare appointments, potentially relieving some fears and uncertainties about symptoms related to memory issues, and in turn** motivating access healthcare.  Narrative  As Cecilia enters her weekly ICT class, she’s greeted warmly by the facilitator. She likes the ICT class because it is always at the same time and place, and it’s a drop-in class so she does not feel any pressure if she cannot attend. Being in her 60s, Cecilia did not grow up using technology but feels eager to learn about it, so she doesn’t get left behind in a digital society. Last year, she was given an old laptop by a friend but wasn’t shown how to use it, so she has been keen to learn how to use it at the ICT class. Cecilia various exercises prepared by the facilitator such as writing emails, typing, creating spreadsheets, and searching for information online. She feels these skills could be very useful if she returns to work in the future and for managing her everyday life when she finds secure accommodation. She likes using her laptop at the ICT class because the facilitator can help if she makes any mistakes, if she forgets how to do something, and because the facilitator encourages her to feel more confident about using the laptop. Cecilia reminiscences about being a student at university back home in Nigeria and attending the ICT classes is reassuring because she has existing skills that she can build on.  Sometimes the facilitator even asks Cecilia to help the other women as she has become increasingly skilled at writing emails and searching for information online. **Recently, she has been using the NHS website which includes videos about how to prepare for various healthcare appointments, examinations, and treatment procedures. She finds that the videos are a useful aid as they provide a step-by-step guide about how to prepare for an appointment and what it may entail. The video settings enable Cecilia to use captions and pause the videos so she can take notes on her smartphone. She has started to use her smartphone to keep a record of her health status, appointments she attends, her medication and treatments. Preparing for healthcare appointments using videos and keeping notes on her smartphone work well for Cecilia as she has been experiencing memory issues lately. She used to feel embarrassed about not remembering, but she appreciated the facilitator’s explanation that computer storage is like a “human brain”. She values the way her laptop and smartphone can assist with her remembering things. Cecilia also likes the security of using Google drive/the Cloud to store and save her “digital memories” even if she loses her laptop or smartphone. Having made notes on her smartphone and searched for information on the NHS website, Cecilia feels more prepared for her upcoming appointment with her GP regarding her memory issues.** |
| --- |

**Supplementary Figure 3.** Continuing to construct the narrative thread by incorporating other participants’ contrasting descriptions and experiences

**Draft 4 of the narrative**

Member checking meetings were performed with all participants (women experiencing homelessness n=9, ICT facilitators n=2) to help to co-construct and refine the narrative, as well as suggesting titles, and identifying pertinent quotes which were incorporated into the draft presented in Supplementary Figure 4. The narrative was presented and further discussed as part of the analysis process in collaboration with an advisory board of women with lived experiences of homelessness (WAB). Revisions are bolded and underlined.

| **“You have to be a digital person now!”**  Summary  Drawing on participant observations from the ICT classes, **narrative three focuses on** learning new ICTs skills, or building on existing competencies, perceived as useful for an envisioned future through and out of homelessness. **ICT classes provide a space for social connection and peer-to-peer learning.** Technology is used as a memory aid and digital health equips the older and middle-aged women experiencing homelessness to become more informed and prepared **for healthcare appointments, easing fears and uncertainties about memory issues, and motivating increased access to healthcare.**  Narrative  As Cecilia enters her weekly drop-in ICT class, she’s greeted warmly by the facilitator. Being in her 60s, Cecilia did not grow up using technology **in Nigeria** but feels eager to learn about it, so she doesn’t get left behind in a digital society. **Cecilia thinks to herself, “But computer classes I like because I need to learn more. You have to be a digital person now!”.** Last year, she was given an old laptop by a friend but wasn’t shown how to use it, so she has been keen to learn how to use it at the ICT class.  With practical support and emotional reassurance from the facilitator, Cecilia practices exercises such as writing emails, typing, creating spreadsheets, and searching for information online. She feels these skills could be very useful if she returns to work in the future and for managing her everyday life when she finds secure accommodation. **Cecilia explains to the facilitator that she enjoys building on her existing skills, “I used to be a secretary, so I know how to do emails and stuff like that.”. However, Cecilia continues that, “… just as I left emails were coming out. I mean I love emails. I don’t like texts. Because I get a lot of people when they contact me, they do text speak. In an email, you know like thanks, T-H-K-S. … That’s not for me. No, when you’re in your 50s or 60s you don’t need to do like the kids of 20.”.**  Sometimes the facilitator asks Cecilia to help the other women as she has become increasingly skilled at writing emails and searching for information online. Recently, she has been using the NHS website which includes videos about how to prepare for various healthcare appointments and what they will entail. Cecilia reads and listens along to the step-by-step video guides, taking notes on her smartphone. She has started to use her smartphone to keep a record of her health status, appointments she attends, her medication and treatments. Preparing for healthcare appointments using videos and keeping notes on her smartphone works well for Cecilia as she has been experiencing memory issues lately. She used to feel embarrassed about not remembering, but she appreciated the facilitator’s explanation that computer storage is like a **“human brain” that stores information.** She values the way her laptop and smartphone can assist with her remembering things, **“my brain is coming back!”. She continued to explain to the facilitator that, “I remember lots of things from my youth but just not the more recent things like this or that button.”.** Having made notes on her smartphone and searched for information on the NHS website, Cecilia feels more prepared for her upcoming appointment regarding her memory issues. |
| --- |

**Supplementary Figure 4.** Final version of narrative three

**Supplementary File 3.** COREQ Checklist.

**COREQ (COnsolidated criteria for REporting Qualitative research) Checklist**

A checklist of items that should be included in reports of qualitative research. You must report the page number in your manuscript where you consider each of the items listed in this checklist. If you have not included this information, either revise your manuscript accordingly before submitting or note N/A.

| **Topic** | **Item No.** | **Guide Questions/Description** | **Reported on**  **Page No.** |
| --- | --- | --- | --- |
| **Domain 1: Research team**  **and reﬂexivity** | | | |
| *Personal characteristics* | | | |
| Interviewer/facilitator | 1 | Which author/s conducted the interview or focus group? | 10-13 |
| Credentials | 2 | What were the researcher’s credentials? E.g. PhD, MD | 15 |
| Occupation | 3 | What was their occupation at the time of the study? | 15 |
| Gender | 4 | Was the researcher male or female? | 15 |
| Experience and training | 5 | What experience or training did the researcher have? | 15 |
| *Relationship with*  *participants* | | | |
| Relationship established | 6 | Was a relationship established prior to study commencement? | 15 |
| Participant knowledge of  the interviewer | 7 | What did the participants know about the researcher? e.g. personal goals, reasons for doing the research | 14-15 |
| Interviewer characteristics | 8 | What characteristics were reported about the inter viewer/facilitator? e.g. Bias, assumptions, reasons and interests in the research topic | 15-16 & 26 |
| **Domain 2: Study design** | | | |
| *Theoretical framework* | | | |
| Methodological orientation and Theory | 9 | What methodological orientation was stated to underpin the study? e.g. grounded theory, discourse analysis, ethnography, phenomenology, content analysis | 9 & 12-16 |
| *Participant selection* | | | |
| Sampling | 10 | How were participants selected? e.g. purposive, convenience, consecutive, snowball | 10-12 |
| Method of approach | 11 | How were participants approached? e.g. face-to-face, telephone, mail, email | 10-12 |
| Sample size | 12 | How many participants were in the study? | 10-12 & Figure 1 |
| Non-participation | 13 | How many people refused to participate or dropped out? Reasons? | Figure 1 |
| *Setting* | | | |
| Setting of data collection | 14 | Where was the data collected? e.g. home, clinic, workplace | 10, 12-13 |
| Presence of non-  participants | 15 | Was anyone else present besides the participants and researchers? | 12-13 |
| Description of sample | 16 | What are the important characteristics of the sample? e.g. demographic  data, date | 11-12 & 14 & Table 1 |
| *Data collection* | | | |
| Interview guide | 17 | Were questions, prompts, guides provided by the authors? Was it pilot tested? | 13 & Supplementary File 1 |
| Repeat interviews | 18 | Were repeat inter views carried out? If yes, how many? | Yes, longitudinal study (Figure 1) |
| Audio/visual recording | 19 | Did the research use audio or visual recording to collect the data? | 12 |
| Field notes | 20 | Were ﬁeld notes made during and/or after the interview or focus group? | 13 & 15-16 |
| Duration | 21 | What was the duration of the inter views or focus group? | 10 & 12 |
| Data saturation | 22 | Was data saturation discussed? | 11 & 26 |
| Transcripts returned | 23 | Were transcripts returned to participants for comment and/or correction? | 13-14 & 26 & Figure 1 |
| **Domain 3: analysis and**  **ﬁndings** | | | |
| *Data analysis* | | | |
| Number of data coders | 24 | How many data coders coded the data? | 3 coders (p. 13) |
| Description of the coding  tree | 25 | Did authors provide a description of the coding tree? | N/A |
| Derivation of themes | 26 | Were themes identified in advance or derived from the data? | 13-14 & Supplementary File 2 |
| Software | 27 | What software, if applicable, was used to manage the data? | 14 |
| Participant checking | 28 | Did participants provide feedback on the findings? | 13-14 & 26 & Figure 1 |
| *Reporting* | | | |
| Quotations presented | 29 | Were participant quotations presented to illustrate the themes/findings?  Was each quotation identified? e.g. participant number | N/A |
| Data and findings consistent | 30 | Was there consistency between the data presented and the findings? | 16-22 & Supplementary File 2 |
| Clarity of major themes | 31 | Were major themes clearly presented in the findings? | 16-22 |
| Clarity of minor themes | 32 | Is there a description of diverse cases or discussion of minor themes? | 22-25 |
